# Errata

**Published:** 1889-05

**Authors:** 


Errata.—For ''setting'' read ''sitting'' on page 450, in Dr. Cupples' remarks; and for ''Dr. Otto Betz,'' in caption of his article, read ''Dr. Odo Betz.''

In the biography of Dr. Swearingen, we omitted to mention that the Louisville Medical College conferred upon him the Honorary Degree in 1887,—an oversight which we much regret.

If there are any other errors don't view us with a critic's eye, for you ought to ''know how it is yourself.''—Ed.

The Austin District Medical Society will meet next on 20th of June prox., in Austin.

